# Personalized cardiometabolic care powered by artificial intelligence

**DOI:** 10.3389/fendo.2025.1593321

**Published:** 2025-05-23

**Authors:** Mansur Shomali, Abhimanyu Kumbara, Janice MacLeod, Anand Iyer

**Affiliations:** ^1^ Welldoc, Inc., Columbia, MD, United States; ^2^ Janice MacLeod Consulting, Glen Burnie, MD, United States

**Keywords:** artificial intelligence, cardiometabolic health, chronic care, data, diabetes, digital health, human-in-the-loop, technologies

## Abstract

Advancements in artificial intelligence (AI) are providing a wealth of opportunities for improving clinical practice and healthcare delivery. It is predicted by AI experts that healthcare will change more in the next decade than it has in the previous century due to the volume and speed of these advancing capabilities. In this paper, we will illustrate the potential value of AI by sharing an example of an AI-powered digital health platform, designed to support people living with chronic cardiometabolic conditions and their care teams. The goal is to transform the care continuum from prevention through diagnosis, treatment, and ongoing management, including efficient acute care interventions when needed. The intent is to shift from reactive to proactive care including preventive-based guidance and interventions. AI-powered health technologies enable true person-centered care (i.e., for N=1), but for entire populations at scale (i.e., N >> 1), shifting the traditional mass generalization paradigm to one of mass customization. We demonstrate how an AI-powered digital health platform: 1) supports early detection and diagnosis of chronic conditions such as diabetes and related cardiometabolic conditions with data and insights; 2) optimizes personalized treatment; 3) tracks progress; 4) provides education; and 5) enables longitudinal behavior change to sustain health. We will present current AI capabilities as well as future considerations for the industry. We will also discuss principles that govern the responsible adoption of AI capabilities in healthcare to complement, not replace, the clinician.

## 1 Introduction – defining AI

While AI is one of the most noted and debated topics in society today, it’s not a novel concept and has been around for many decades. Of note is the period from 1950 to 1956, where Alan Turing famously published “Computer Machinery and Intelligence” which proposed a test of machine intelligence called The Imitation Game. In 1952, a computer scientist named Arthur Samuel developed a program to play checkers, which was the first to ever learn the game independently. AI has been universally defined as any technique that enables computers to mimic human intelligence using a series of logical constructs, including if-then rules, decision trees, machine learning and deep learning (1). Interestingly, the American Medical Association also uses the term “augmented intelligence”, focusing on AI’s assistive role and emphasizing the fact that AI enhances human intelligence rather than replacing it (2). We now take a closer look at these forms of AI (see **Supplementary Figure S1**).

Rule-based artificial intelligence (AI) is a type of AI that uses predefined rules - often derived from clinical evidence-based guidelines for healthcare applications - to process data and make decisions. It’s a static approach that is well-suited for environments where rules and outcomes are traceable and don’t change. An example of a rule-based AI would be an insulin calculator that uses an insulin-to-carbohydrate ratio and a correction factor to recommend an insulin bolus dose based on a defined carbohydrate intake and a healthcare provider’s prescription for how the insulin should be administered. The output of such a model will always be the same for the same permutations of input variables and will not evolve or change over time. The benefit of such an approach is that it is testable, and can be assessed confidently for risk, which will not evolve or change as it can be assessed for every viable permutation of input variables to the model (1).

Machine Learning (ML) is a type of AI that enables systems to learn from data, identify patterns, and make decisions with varying degrees of human intervention. There are different types of machine learning:

- Supervised learning: Algorithms are trained on labeled data and direct supervision by a human.
- Unsupervised learning: A type of machine learning that learns from data without human supervision. Unlike supervised learning, unsupervised machine learning models are given unlabeled data and allowed to discover patterns and insights without any explicit guidance or instruction.
- Reinforcement learning: Combining the above approaches, with human intervention, to allow the system to learn through trial and error, receiving rewards for correct actions.

A benefit of such an approach is that the output of the model can adapt and change to variations in the input variables. In essence, the model learns over time, and becomes more accurate and in the case of health interventions, more personalized, an important step in the journey towards mass customization (3).

Deep learning (DL) is one type of ML involving neural networks with many layers, enabling high-level data abstraction and pattern recognition. DL can be used for advanced image analysis for pathology and radiology, for speech recognition and natural language processing applications, and for genomic data analysis for personalized medicine. While ML typically relies on thousands of data points, DL uses techniques that accommodate millions of data points. ML algorithms usually perform well with relatively small datasets. DL requires large amounts of data to understand and perform better than traditional machine learning algorithms.

Generative AI is a broad category for a type of AI, referring to any artificial intelligence that can create original content. Generative AI applications are built on underlying foundational AI models, such as large language models (LLM). LLMs are the text-generating basis of generative AI. Generative AI models work by using a combination of attention layers and neural networks to extract semantic, contextual, and grammatical patterns from large volumes of text training data, which can then generate new content (4).

All of these forms of AI can manifest within the healthcare system. Each can play a different role that is accompanied by varying degrees of risk and intended use; i.e., is the AI intended to take a current process or system and make it more efficient (i.e., better, faster, less expensive, more scalable, etc.) or is it to deliver something novel that could otherwise not be achieved (5–7). We discuss this AI application framework later in the paper.

## 2 An AI architecture to transform the care continuum

**Figure 1:**
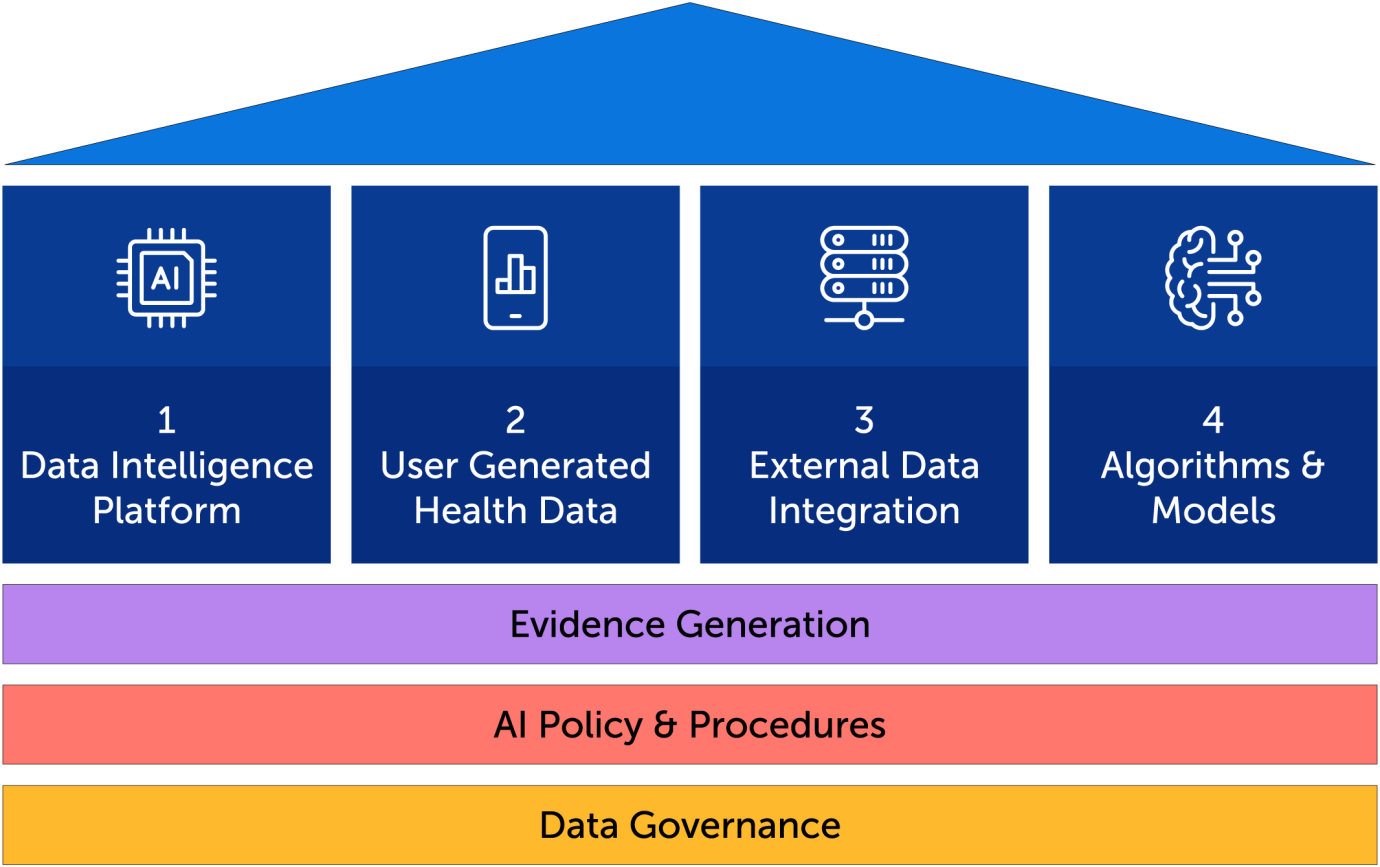
The foundational architecture for an AI-powered digital health platform.

In order to achieve care transformation goals through AI-powered technology, a solid foundation is needed which includes data governance, AI policy and procedures and evidence generation. From this foundation, four pillars rise, as illustrated in **Figure 1**: 1) A data intelligence platform, 2) user-generated health data through a digital health solution, 3) the integration of external health data sets from data shared by clients and external sources, 4) the underlying AI algorithms and models including model quality and evaluation.

### 2.1 The data intelligence platform

The data intelligence platform includes the data infrastructure, data engineering, data analytics, data visualization, and AI modeling that collectively enable the conversion of raw data into insights and value that can be used for different purposes with different stakeholders. While the data intelligence platform’s “DNA” is characterized by the elements listed above, it can best be functionally described in four sections:

1. Inputs. The inputs to the data intelligence platform are data, which can come from a variety of sources. These data are structured, semi-structured, and unstructured, and can be derived from biometric and non-biometric data sources that feed into a digital health platform or app. Examples of biometric sources are sensors, such as continuous glucose monitoring systems (CGMs), wearables and in the future, a plethora of planned non-invasive physiologic sensing devices. A digital health app can additionally collect individual level data from the user around symptoms, medications, psychosocial, food, activity, sleep, and individual health and laboratory testing results and provides this data, including summarized insights that are made available in a clinical portal. These types of data constitute the principal data sources and using a water analogy, could be described as different rivers emptying large volumes of water into a larger lake - in this case, a lake or lakehouse of data. But, the sources are not just limited to these larger volumes of data. Into this large body of water, smaller streams can also trickle in additional data from external sources (e.g., Firebase, Bitly, Sendbird, Apple iTunes and Google Play app store, Google Analytics, and Ring Central, etc.). To summarize, the inputs are data that can be associated with the following four categories.
  - Digital health app engagement data: This is person-entered data into a digital health app (e.g., daily blood pressure and weight values, images of food consumed, surveys and health literacy courses completed, etc.).
  - Event and communications data: This is ancillary data that surrounds the use of the app, related to external SMS, email and other notification data.
  - External member data: This can be lab, pharmacy, genomic or other data associated with the member or members.
  - App activation data: This is data related to the activation process, and may provide “string of pearls” insight into the activation journey a member is taking.
2. Extract-Transform-Load (ETL) Processes: The “extract-transform-load” is a process that combines data from the multiple input sources above into a centralized storage repository for analysis and storage. It’s a common way to prepare data for machine learning, data analytics, and business intelligence. It will involve steps that include data organization, data cleansing, data definition capture (i.e., data dictionary preparation) among other data pre-processing steps and techniques. It may also involve translation, which would be format manipulation to allow for downstream processing of the data in a unified manner.
3. Data Lake: This is the key power of the data intelligence platform, and can be implemented through a variety of platform tools such as Databricks, Azure Synapse, Amazon Redshift, etc. These platforms allow for efficient storage and partitioning of data into raw tables (i.e., bronze-layer), pre-processed data (e.g., silver-layer) and aggregated data (e.g., gold-layer). For example, while raw continuous glucose monitoring (CGM) data may be brought into the bronze layer, the calculation of the next level of metrics such as time in range, time above range, estimated average glucose and glucose variability would occur and be housed in the silver layer for easy and efficient use. Data architecture is paramount in order to optimize separability of data in a manner that is governed by data storage size, frequency of access, type of data, location of data, etc. For example, housing data together that is frequently accessed allows for storage capacity to be added only where needed due to frequency of use, vs. a more “brute force” way of adding storage capacity, perhaps to where it is not necessary, thus leading to possible excess costs due to underutilized capacity.
4. Data Sharing and Analytics: This section addresses an important part of the data intelligence platform, addressing “what can one do with the data?” Inherent in this section is advanced reporting and visualization of the data, key trends over time, key comparisons across different cohorts, etc. Also, the ability to share data with external parties - under proper consent, privacy, legal and security procedures and policies - in multiple formats is paramount; data sharing allows entities who are downstream in the data value chain to extract value from data at all levels.

### 2.2 User-generated health data

A digital health app’s advanced AI-Engine connects and correlates among various dimensions of health (MEDALS; Medications, Education, Diet, Activity, Labs, Symptoms and Surveys) which informs personalized guidance and coaching messages. This is the fundamental architecture for Welldoc’s digital health apps. Engagement with any digital health solution is required for it to deliver its intended purpose to an individual with a chronic condition such as diabetes. In diabetes, it is well established that optimal glucose management requires: 1) an understanding of an individual’s glucose levels in real-time; and 2) diligent self-management of key behaviors including the Association of Diabetes Care and Education Specialists (ADCES) ADCES7 Self-Care behaviors (eating healthy, being active, taking medications, monitoring, problem solving, reducing risks and healthy coping (8)). The combination of CGM which can provide dense glucose data, and AI-driven, regulated digital health solutions, which capture the user engagement data, helps to address these two factors as illustrated in **Supplementary Figure S2** showing examples from Welldoc’s digital health application.

Welldoc conducted analysis to see if engaging with the combined CGM+AI-driven digital coaching influenced self-management behaviors. Data were reviewed from individuals living with type 2 diabetes enrolled in an employer program that provided a continuous glucose monitoring system and the digital health platform to each participant. Participant engagement with the digital health solution was examined across two cohorts: Cohort 1 (n=37) used CGM continuously for 24 weeks; Cohort 2 (n=55) used CGM intermittently. Counts of specific feature use of the digital health solution that support ADCES7 self-care behaviors, including food, medication tracking, activity, sleep, blood pressure, and weight were tabulated. At baseline: 56% of CGM users were male; 80% were between 40–64 years years-old; mean baseline A1C was 9.5%; most participants were not prescribed insulin. It was found that use of CGM+AI-driven Digital Coaching can help individuals with type 2 diabetes regardless of their therapy type to improve ADCES7 self-care behaviors engagement. Engagement with the digital health solution was significantly greater for the continuous use cohort in the first 12 weeks (37.1 Weekly Average Engagements versus 18.8; p=0.00005) (9). Clinicians should consider how CGM wear time influences self-care behaviors to coach and treat individuals with diabetes. Further study of the integration of emerging real-time data sources into digital health offerings will inform the continued evolution of digital solutions and the support they provide for individuals managing their chronic care journey.

### 2.3 External data integration

As medical knowledge grows, the volume and types of health data expands and as technologies advance, insights from the data can support clinical workflows and decision-making. This data can include (but is not limited to) claims data, genomic data, ethnographic data, social determinants of health (SDOH) data, survey data, geolocation data, zip code data, and so forth (see **Supplementary Figure S3**) (10). Diabetes is a condition in which ever-advancing technologies have served to create a digital diabetes ecosystem of connected technologies including CGMs, insulin pumps including automated insulin delivery (AID), and smart insulin pens along with numerous health and lifestyle apps (11, 12). These technologies support people living with diabetes in their daily self-management while gathering valuable self-management and lifestyle data that can be transmitted and analyzed. With the ongoing advances in AI, it is possible to integrate current technologies with precision medicine learnings into AI software including physiological data, psychological data based on validated instruments, e.g. diabetes distress, and reported or tracked health behaviors such as taking medications, being active and healthy eating. AI can then provide precise, holistic therapy recommendations individualized to each person (11).

Two factors need to be considered with regard to the potentially large volume of external data: one, how clinicians will access these data; two, how will clinicians utilize these data effectively in their work flows. Typically, data from glucose sensors, insulin pumps, and food and activity trackers could be viewed via a web portal. The clinician would have to log in with a username and password and then locate the patient and then view and manually transfer that data to the electronic health record (EHR). This clearly is cumbersome and takes clinicians away from their optimal work flow within the EHR. In the near future, most web portals will be replaced with direct data integration with the EHR. The data should always be synced in the electronic chart of the patient and can be added to the encounter documentation easily. In addition, direct integration would facilitate virtual visits and remote monitoring.

Regarding the second point, access to person-generated data continuously can be overwhelming and may increase burden on clinicians. AI systems should be able to summarize the data and produce correlative insights that would give clinicians the actionable information that they need. Alerts could be generated only when clinicians need to be aware of critical results. Such algorithms would thus free up clinicians for higher cognitive tasks. When computers do what computers do the best, clinicians will be freed up to be more humanistic caregivers, engendering trust and empathy with their patients.

In a recent study, clinicians were given a task to make diagnoses and demonstrate clinical reasoning when given standardized patient vignettes. The clinicians were randomized to the use of an AI for clinical decision support or to the use of traditional medical references. The results surprisingly demonstrated no differences in the clinical reasoning scores between the two groups. In fact, the AI by itself performed better than the clinicians with the AI. This suggests that AI tools cannot be given to clinicians without training. Clinicians must learn to use AI optimally to get the best results (13).

### 2.4 Algorithms and models

Since clinical AI systems are developed on vast amounts of real-world health data, the corresponding labels and data quality will influence an AI model’s performance. Potential challenges associated with the quality of data can include: 1) poor quality of the data themselves (e.g., blurred images, intermittent data from a CGM, etc.); 2) incorrect data labels; and 3) insufficient data, with only a small portion of the data being labeled (14). AI algorithms must be trained on fair datasets that accurately represent social, environmental, and economic factors which influence health. Otherwise, they may amplify implicit bias and discrimination if trained on data that reflect the health-care disparities experienced by groups defined by race, ethnicity, gender, sexual orientation, socioeconomic status, or geographic location (15). The STANDING Together consensus workgroup has published recommendations, through an international consensus process, which provide guidance on transparency around who is represented in the data, how people are represented, and how data is used when developing AI technologies for healthcare (16). Choosing an appropriate AI model, based on the use case, is also important. Some AI models, like Generative AI, are resource intensive (e.g., require significant computing power), hence picking the right model is important for successful implementation of AI models in practice.

### 2.5 Evidence generation

Digital health is significantly altering the way researchers approach research as it is enabling the collection of real-world data (RWD). Large-scale, continuous data from diverse patient populations through wearable devices, mobile apps, and integrated electronic health records (EHRs), allows for more representative insights into treatment effectiveness compared to that with more controlled settings of traditional randomized controlled trials (RCTs), which can be less generalizable to real-world practice (17). By leveraging data from large patient populations using digital health platforms, researchers can study diverse groups and identify patterns that may not be apparent in smaller RCTs. Digital health technologies empower individuals to actively participate in research by self-reporting data through apps and wearables, leading to more person-centered insights and providing a richer picture of health outcomes beyond clinical visits. Continuous data collection allows for monitoring treatment effects in real-time, enabling rapid adjustments and better understanding of treatment dynamics. Utilizing existing digital health infrastructure can be more cost-effective than conducting traditional RCTs, especially for long-term studies.

While digital health is enabling researchers to move beyond the limitations of RCTs by providing access to rich, real-world data, there are challenges associated with using digital health for real-world evidence that must be considered. Data collected through digital platforms can be incomplete, inconsistent, or biased due to user behavior and device limitations. Ensuring patient privacy and data security is critical when collecting large amounts of personal health information. Analyzing large, diverse datasets from digital health sources requires advanced statistical methods and expertise. Lack of standardized data collection protocols across different digital health platforms can hinder data comparability.

Despite research challenges there is some evidence of the potential of digital health to improve clinical outcomes in cardiometabolic conditions. A cluster-randomized trial of an early digital health tool powered by rules-based AI enrolled 163 patients with type 2 diabetes across 26 primary care practices. Results showed a mean decline in A1C of 1.9% in the maximal treatment group (mobile- and web-based self-management patient coaching system and provider decision support) compared to 0.7% in the usual care group, a difference of 1.2% (P<0.001). There were no appreciable differences observed between groups for patient-reported diabetes distress, depression, diabetes symptoms, or blood pressure and lipid levels (all P>0.05) (18).

More recently a study sought to demonstrate the safety of a CGM-informed insulin bolus calculator that applies trend arrow and exercise adjustments to bolus insulin dose recommendations and provides real-time coaching through an AI-powered digital health tool. Fifty-four participants with either type 1and type 2 diabetes and using CGM were enrolled in a 30-day prospective clinical trial conducted at two research sites. Participants used the mobile application to monitor their CGM data and calculate insulin doses. Time in Range (TIR) during the prospective 30-day period was compared to that in 30 days of baseline data. Participants’ TIR improved from 68.4% to 71.8% (N=54, P=0.013) with no increase in time below 70 mg/dL. Researchers noted that individuals with type 2 diabetes had a greater increase in TIR (74.3% to 81.4%) than those with type 1 diabetes; 64.4% to 65.2%). In users with at least moderate diabetes distress at baseline, there was a significant reduction in diabetes distress at study completion. Use of a novel CGM-informed insulin bolus calculator by individuals with diabetes was associated with significant improvement in TIR without an increase in hypoglycemia or diabetes distress (19).

In a review of key papers (written between July 1, 2021 and June 30, 2022), addressing both digital therapeutics and digital care solutions in the prevention and treatment of diabetes Clements et al. found a focus on translating evidence-based diabetes prevention programs into validated digital delivery formats and on remote physiological monitoring as the use of connected technologies monitoring aspects of metabolic health expands (20). One of the studies reviewed was a randomized controlled trial of an interactive smartphone app-based lifestyle intervention for weight loss conducted in 28 adults with a body mass index between 25–42 kg/m^2^. Subjects were randomized to either a conventional weight loss program or to the smart phone digital health intervention. This app-based electronically delivered lifestyle intervention produced statistically significant, clinically meaningful weight loss and improved metabolic health at 6 months (21). Engagement with the intervention correlated strongly with weight loss. Researchers note that larger and longer studies of this intervention are needed.

Another study reviewed was a secondary analysis of retrospective data of adults with prediabetes who were enrolled in a digital diabetes prevention program which incorporates interactive mobile computing, remote monitoring, and evidence-based curriculum, behavior tracking tools, health coaching, and online peer support to prevent or delay type 2 diabetes. The sample (N=1095) included people with prediabetes who completed at least 9 months of the digital prediabetes program. Participants were 67.7% (n=741) female, with a mean age of 53.6 (SD 9.75) years. After 12 months, participants decreased their weight by an average of 10.9 lbs (5.5%; *P*<.001) and increased their physical activity by 91.2 (*P*<.001) minutes. These results suggest that the digital prediabetes program is effective at preventing type 2 diabetes through a significant reduction in body weight and increase of physical activity. A prospective randomized controlled clinical study is needed to validate these findings (22).

Evidence generation is not only important for clinical outcomes, but for economic outcomes as well. Implementation and scaling of AI technology is associated with high costs. Studies should examine the cost-effectiveness, potential savings, and the economic feasibility of any novel AI solutions across healthcare systems. Health economic analyses should assess the value for specific AI interventions and can thus support the optimal allocation of health system resources (23).

### 2.6 AI policy and procedures

Elbert Hubbard once said “The world is moving so fast these days that the person who says it can’t be done is generally interrupted by someone doing it!” (24). Indeed, this can be said of the guardrails that are attempting to be put into place by esteemed organizations, such as the FDA, White House and most recently and perhaps most comprehensively, the United Nations. Recent developments in AI policy and procedures for healthcare in the US focus on balancing innovation and speed with patient safety and ethical considerations.

The FDA is actively working on frameworks for regulating AI-based medical devices and has issued a guidance document, emphasizing the need for transparency and validation. Discussions are ongoing regarding the use of AI in diagnostics, treatment planning, and administrative tasks, with concerns about bias, privacy, and the potential impact on healthcare professionals (25). Organizations like the National Academy of Medicine are promoting guidelines for responsible AI implementation in healthcare, while various stakeholders are collaborating to address challenges and ensure AI benefits all patients equitably (26, 27).

The United Nations issued “Governing AI for Humanity”, in September 2024, as a collaborative, international effort to undertake analysis and advance recommendations for the international governance of artificial intelligence (28). In this report, the point is made that “There is, today, a global governance deficit with respect to AI. Despite much discussion of ethics and principles, the patchwork of norms and institutions is still nascent and full of gaps. Accountability is often notable for its absence, including for deploying non-explainable AI systems that impact others. Compliance often rests on voluntarism; practice belies rhetoric.”

Much work remains to be done in ensuring diligent process and policy for the safe, judicious and valuable integration of AI into healthcare delivery as we know it today.

### 2.7 Data governance

We are committed to understanding how AI can be leveraged to power better connected care. This work is complex, requiring diligence in operationalizing extensive and diverse data sets, clinical evidence, data governance, interoperability and a data intelligence platform that ensures privacy, security and scalable application in real-world settings. Databricks, with its Unity Catalog, is an example of a platform that gives superior centralized data governance capabilities. Unity Catalog allows seamless governance of various data sources to be shared with enterprise clients. Fairness and bias testing allows for inclusive models to be built and operationalized. Good data governance ensures the mitigation of potential harm and helps to build and maintain public trust.

## 3 Combining architecture elements to form an AI strategy: balancing risk and value

Armed with data governance, a data intelligence platform, person-generated health data and external data sets, it now remains to build an AI strategy by organizing potential use case opportunities into a framework defined by two principal dimensions: Risk (high or low) and value/AI intent (operational efficiencies or innovation/new capabilities) as illustrated in **Supplementary Figure S4**. Use cases or features that utilize each type of AI (Rules-based, ML, DL, Generative AI) can manifest in any of the four quadrants. In the context of software as a medical device (SaMD), the categorization of risk is nearly synonymous with unregulated vs. regulated features and functionality.

### 3.1 A “Crawl-Walk-Run” approach

The opportunities in digital health created by AI can be implemented in stages - aligned with the evolution of AI itself - as outlined below, with inherent value-risk increasing across the stages. At each stage there are constraints and considerations including: regulatory oversight; the extent and size of data sets required for learning; comprehensive research and algorithm development; and robust testing and monitoring. As an illustrative example, for a digital health solution that utilizes AI to coach individuals who suffer from a chronic condition on how to best manage their health based on data they enter into the solution, the evolution can be as follows:

- Stage 1: Rules-based: Objective: Static digital coaching and insights based on clinical guidelines.
- Stage 2: Dynamic: Objective: Enhanced digital coaching and insights based on an individual’s data, to drive more precision and personalization within what the clinical guidelines allow for.
- Stage 3: Adaptive: Objective: Organic (i.e., living) optimization of coaching and automation of actions and insights.

To orchestrate and implement a successful AI strategy, many factors need to be taken into consideration:

- Availability of representative data to train AI models: There is no model without data. A typical model training approach will partition the available data in a 70:30 ratio; that is, 70% of data is used for training the model and 30% for testing the model. There are many variants for how models can be trained, but it’s necessary to have the right data, and plenty of it.
- Discipline in training and testing when it comes to bias, fairness, and toxicity. Rigorous training and testing methods, which invoke a series of metrics such as AUC (area under the curve), ROC (receiver operating characteristic curve), accuracy and MSE (mean square error) can be used to ensure that model training and testing iterations strive to optimize the solution to the problem in consideration.
- Models that are trained on owned data to enable traceability and data provenance. One of the core issues, asked especially in regulated medical device use of AI, is regarding data provenance; that is, a documented trail that tracks the history of data, including its origin, changes, and processes that are used to manipulate it. Metrics on accuracy will need to be traced back to data sources, to ensure that models don’t unfairly classify outcomes, or classify outcomes with bias. With increased emphasis on inclusiveness, ensuring data provenance becomes critical to successful model development and deployment.
- Periodic retraining and re-testing of models to drive continuous accuracy improvement. The concept of data drift is important to embrace. Will a model, trained on the current year’s data, be accurate in 5 years, with advancements in drug therapies, coaching models, real world insights, etc.? Will models behave the same way over time? It is important to ensure that models are periodically re-trained to accommodate for data drift over time.
- Integrated algorithm development to minimize data processing and infrastructure costs. It is said that less than 10% of Fortune 500 companies have a data lakehouse strategy (Accenture), which is a key element required to even contemplate an AI strategy. Being able to use one’s own data, within the data intelligence platform will not only benefit issues such as provenance but will also contribute to overall lower processing and infrastructure cost (29).

And, let us not forget some of the foundational enablers for a successful AI strategy, which can include basic macro-economic factors such as access to digital technology, digital and Internet connectivity and technology literacy (30).

## 4 Looking to the future

**Figure 2:**
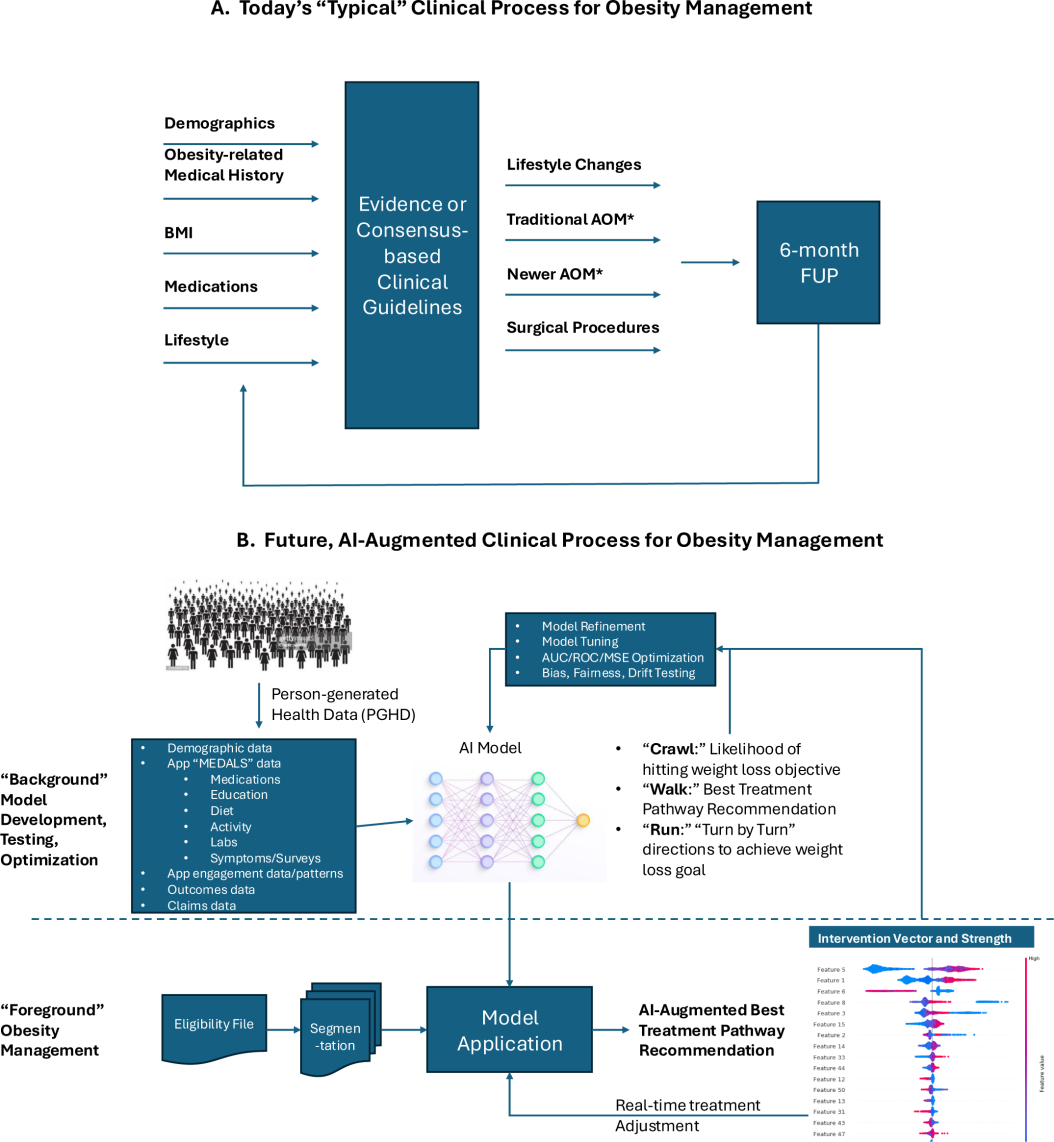
Example of a future AI-enabled weight management program. **(A)** The traditional approach to weight management includes a clinical assessment, implementation of a treatment plan based on standards of care, and a follow-up assessment, at which point the treatment plan can be adjusted. **(B)** The AI-enabled program also includes a clinical assessment, but then the model (as prescribed by the clinician) can make real-time adjustments in the care plan based on person-generated health data.

The use of AI in healthcare is increasing, contributing to improved diagnostic accuracy, optimized treatment plans, and improved clinical outcomes. The rapid evolution of AI, especially generative AI and the large language models (LLMs) have set off discussions about the potential impact of AI on the healthcare industry and the evolving role of the healthcare provider or clinician in various disciplines. The question is frequently raised, “will AI replace clinicians” or “will clinicians who use AI replace clinicians who do not use AI?” (7, 31). AI is best used when it augments or complements the clinician’s own judgment and scientific training versus replacing them, thereby combining the cognitive strengths of the clinician with the analytical capabilities of AI. This concept has been described as a human-in-the-loop approach to ensure that AI systems are guided and supervised by human expertise to maintain safety and quality in healthcare delivery (32). This approach is resulting in a paradigm shift in healthcare by using AI to complement and enhance the skills of clinicians, ultimately leading to improved service, quality, clinical outcomes, and healthcare system efficiencies (see example in **Figure 2**).

Healthcare organizations will increasingly become responsible for providing AI tools that have undergone rigorous evaluation and validation to ensure safety and effectiveness for clinical practice (e.g., regulatory review and clearance). An example of this could be the integration of augmented reality into chronic care management. The potential impact that Augmented Reality (AR) may have on a condition such as diabetes can be profound (33). An AR system may push the envelope on lifestyle-enhancing benefits for users, ultimately allowing them to make better choices. AR glasses/devices could be used to visualize real-time changes in blood glucose levels based on dietary choices, for example. For prognostic or feedback purposes, data on glucose, insulin sensitivity, diet, and activity could all be incorporated by the system. Thus, AR, large language models, and machine learning, when integrated into one platform, have the potential to offer personalized, intelligent, and interactive experiences not fathomable today.

Finally, there is a growing need to develop education and training on the fundamentals of AI and its effective implementation in clinical practice and AI-supported healthcare delivery, and such training will undoubtedly take on a grass-roots flavor and end up in medical school training curricula. Critical to success will be the thoughtful integration of AI-powered tools into the clinical care process, a focus on user engagement, decision support truly anchored to data, and incentives focused on health outcomes and prevention.

## 5 Conclusion

Leveraging AI to fix a failing healthcare system will clearly require interdisciplinary work by clinicians, healthcare executives, medical device companies, data scientists, ethicists, and public health professionals, among others. It is up to clinicians to recognize their important role in responsibly leveraging AI to fix a failing healthcare system. While the potential impact of AI on healthcare is still being determined, ongoing digital innovation is ensuring an ever-growing toolbox of new solutions giving momentum to AI interest and eventual adoption. Regulatory guidance will need to accommodate the rapid and ongoing iteration of digital health tools including new ways of monitoring health data. We are hopeful that by describing the overarching architecture of our AI-powered digital health solution, that we will serve to guide clinicians, healthcare systems, researchers, payers, and other stakeholders in responsible adoption of AI technologies that are rigorously developed and maintained to ensure effectiveness and safety in the more efficient delivery of healthcare, particularly for those with complex chronic conditions.

## Data Availability

The original contributions presented in the study are included in the article/**Supplementary Material**. Further inquiries can be directed to the corresponding author.

## Supplementary material

Supplementary Figure 1 Types of Artificial Intelligence (Adapted from Bellini et al, 2022).

Supplementary Figure 2 User-generated health data and digital coaching.

Supplementary Figure 3 Wearable/connectables technologies and external data sources.

Supplementary Figure 4 Balancing AI intent with risk.

## References

[1] Bellini V Cascella M Cutugno F Russo M Lanza R Compagnone C. Understanding basic principles of artificial intelligence: a practical guide for intensivists. Acta BioMed. (2022) 93:e2022297. doi: 10.23750/abm.v93i5.13626 36300214 PMC9686179

[2] AMA. The future of health: The emerging landscape of augmented intelligence in healthcare. Available online at: https://www.ama-assn.org/practice-management/digital/ama-future-health-emerging-landscape-augmented-intelligence-health-care (Accessed September 30, 2024).

[3] Jacobs PG Herrero P Facchinetti A Kovatchev B Breton MD Cinar A. Artificial intelligence and machine learning for improving glycemic control in diabetes: best practices, pitfalls, and opportunities. IEEE Rev BioMed Eng. (2024) 17:19–41. doi: 10.1109/RBME.2023.3331297 37943654

[4] Sheng B Guan Z Lim LL Jiang Z Mathloudakis N Li J. Large language models for diabetes care: potentials and prospects. Sci Bull. (2024). doi: 10.1016/j.scib.2024.01.004 38220476

[5] Lawry T. Hacking healthcare how AI and the intelligence revolution will reboot an ailing system. New York, New York: Routledge Taylor & Francis Group A Productivity Press Book (2023).

[6] Chaudhri A Lillrank P. Mass personalization in healthcare: insights and future research directions. J Adv Manage Res. (2013). doi: 10.1108/JAMR-05-2013-0033

[7] Midyett LK. One size fits all versus individualized medicine in type 1 diabetes. Diabetes Technol Thera. (2023) 25:S42–7. doi: 10.1089/dia.2023.0109 37306440

[8] Kolb L. An effective model of diabetes care and education: The ADCES7 Self-Care Behaviors™. Sci Diabetes Self-Management Care. (2021) 47:30–53. doi: 10.1177/0145721720978154 34078208

[9] Kumbara AB Iyer AK Green CR Jepso LH Leone K Layne JE. Impact of a combined continuous glucose monitoring-digital health solution on glucose metrics and self-management behavior for adults with type 2 diabetes: real-world observational study. JMIR Diabetes. (2023) 8:347638. doi: 10.2196/47638 PMC10520761 37590491

[10] Subbiah V. The next generation of evidence-based medicine. Nat Med. (2023) 29:49–58. doi: 10.1038/s41591-022-02160-z 36646803

[11] Levine B Close KL Gabbay RA. Reviewing U.S. connected diabetes care: the newest member of the team. Diabetes Technol Thera. (2020) 22. doi: 10.1089/dia/2019.0273 31483160

[12] Phillip M Bergenstal RM Close KL Danne T Garg SK Heinemannn L. The digital/virtual diabetes clinic: the future is now—recommendations from an international panel on diabetes digital technologies introduction. Diabetes Technol Thera. (2021) 23. doi: 10.1089/dia.2020.0375 PMC8098767 32905711

[13] Goh E Gallo R Hom J Strong E Weng Y Kerman H. Large language model influence on diagnostic reasoning: A randomized clinical trial. JAMA Netw Open. (2024) 7:e2440969. doi: 10.1001/jamanetworkopen.2024.40969 39466245 PMC11519755

[14] Bitkina OVI Park J Kim HK. Application of artificial intelligence in medical technologies: a systematic review of main trends. Digital Health. (2023) 9:1–15. doi: 10.1177/20552076231189331 PMC10359663 37485326

[15] Guan Z Li H Liu R Cai C Liu Y Li J. Artificial intelligence in diabetes management: Advancements, opportunities, and challenges. Cell Rep Medicine. (2023). doi: 10.1016/j.xcrm.2023.101213 PMC10591058 37788667

[16] Alderman JE Palmer J Laws E McCradden MD Johan O Marzyeh G. Tackling algorithmic bias and promoting transparency in health datasets: the Standing Together consensus recommendations. N Engl J Med AI. (2025) 2. doi: 10.1056/AIp2401088 PMC11668905 39701919

[17] Kim M Patrick K Nebeker C Godino J Stein S Klasnja P. The digital therapeutics real-world evidence framework: an approach for guiding evidence-based digital therapeutics design, development, testing, and monitoring. J Med Internet Res. (2024) 26:e49208. doi: 10.2196/49208 38441954 PMC10951831

[18] Quinn CC Shardell MD Terrin ML Barr EA Ballews SH Gruber-Bablini AL. Cluster-randomized trial of a mobile phone personalized behavioral intervention for blood glucose control. Diabetes Care. (2011) 34:1934–42. doi: 10.2337/dc11-0366 PMC3161305 21788632

[19] Shomali M Kelly C Kumbara A Iyer A Park J Aleppo G. Safety of a novel continuous glucose monitoring–informed insulin bolus calculator mobile application for people with type 1 or type 2 diabetes. Diabetes Spectr. (2024), ds240032. doi: 10.2337/ds24-0032 PMC12078995 40386812

[20] Clements M Kaufman N Mel E. Using Digital health technology to prevent and treat diabetes. Diabetes Technol Thera. (2023) 25:S90–S108. doi: 10.1089/dia.2023.2506 36802181

[21] Vaz CL Carnes N Pausti B Zhao H Williams KJ. A randomized controlled trial of an innovative, user-friendly, interactive smartphone app-based lifestyle intervention for weight loss. Obes Sci Pract. (2021) 7:555–68. doi: 10.1002/osp4.v7.5 PMC8488442 34631134

[22] Batten R Alwashmi MF Mugford G Narrio M Besner A Gao Z. A 12-month follow-up of the effects of a digital diabetes prevention program (VP Transform for Prediabetes) on weight and physical activity among adults with prediabetes: secondary analysis. JMIR Diabetes. (2022) 7:e23243. doi: 10.2196/23243 35029532 PMC8800085

[23] Turner HC Archer RA Downey LE Isaranuwatchai W Chalkidou K Jit M. An introduction to the main types of economic evaluations used for informing priority setting and resource allocation in healthcare: Key features, uses, and limitations. Front Public Health. (2021) 25:722927. doi: 10.3389/fpubh.2021.722927 PMC8424074 34513790

[24] Esar E. The treasury of humorous quotations. New York, New York. (1951). p. 103.

[25] New York, New York. Available online at: https://www.fda.gov/regulatory-information/search-fda-guidance-documents/considerations-use-artificial-intelligence-support-regulatory-decision-making-drug-and-biological (Accessed February 6, 2025).

[26] Health care artificial intelligence code of conduct. Natl Acad Med. Available online at: https://nam.edu/news-and-insights/nam-leadership-consortium-collaborates-with-leading-health-tech-research-and-bioethics-organizations-to-develop-health-care-ai-code-of-conduct/.

[27] Coalition for health AI. Available online at: https://chai.org/ (Accessed February 6, 2025).

[28] United Nations AI Advisory Board. Governing AI for humanity final report. September 2024. Governing AI for Humanity - Final Report. (Accessed February 6, 2025).

[29] Pala SK. Databricks Analytics: empowering data processing, machine learning and real-time analytics. EDUZONE: Int Peer Reviewed/Refereed Multidiscip J (EIPRMJ). (2021) 10(1). Available online at: www.eduzonejournal.com (Accessed September 30, 2024).

[30] Benda NC Veinot TC Sieck CJ Ancker JS. Broadband internet access is a social determinant of health! Am J Public Health. (2020) 110:1123–5. doi: 10.2105/AJPH.2020.305784 PMC7349425 32639914

[31] Sezgin E. Artificial intelligence in healthcare: complementing, not replacing, doctors and healthcare providers. Digital Health. (2023) 9:1–5. doi: 10.1177/20552076231186520 PMC10328041 37426593

[32] Mosqueira-Rey E Hernández-Pereira E. Alonso-Ríos, D. et al. Human-in-the-loop machine learning: a state of the art. Artif Intell Rev. (2023) 56:3005–54. doi: 10.1007/s10462-022-10246-w

[33] Eckert M Volmerg JS. & Friedrich, C. M. Augmented reality in medicine: systematic and bibliographic review. JMIR Mhealth Uhealth. (2019) 7:e10967. doi: 10.2196/10967 31025950 PMC6658230

