# Supplementary figures and images for: Personalized cardiometabolic care powered by artificial intelligence
Source: Front Endocrinol (Lausanne). 2025 May 23;16:1593321. doi: 10.3389/fendo.2025.1593321 (PMC12142687; doi:10.3389/fendo.2025.1593321)

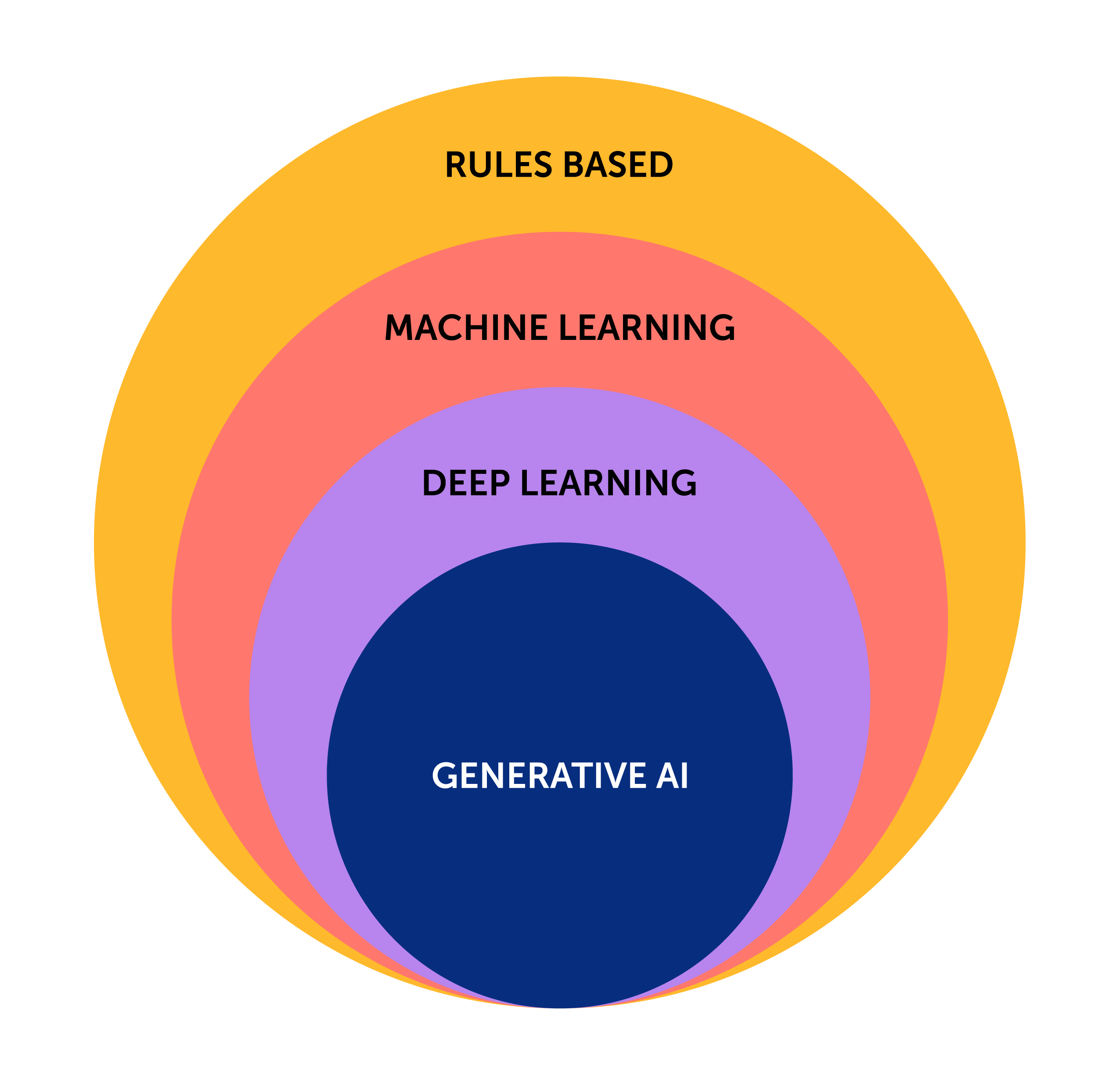

Supplement: Supplementary Figure 1 — Types of Artificial Intelligence (Adapted from Bellini et al, 2022). [file Image1.tif]

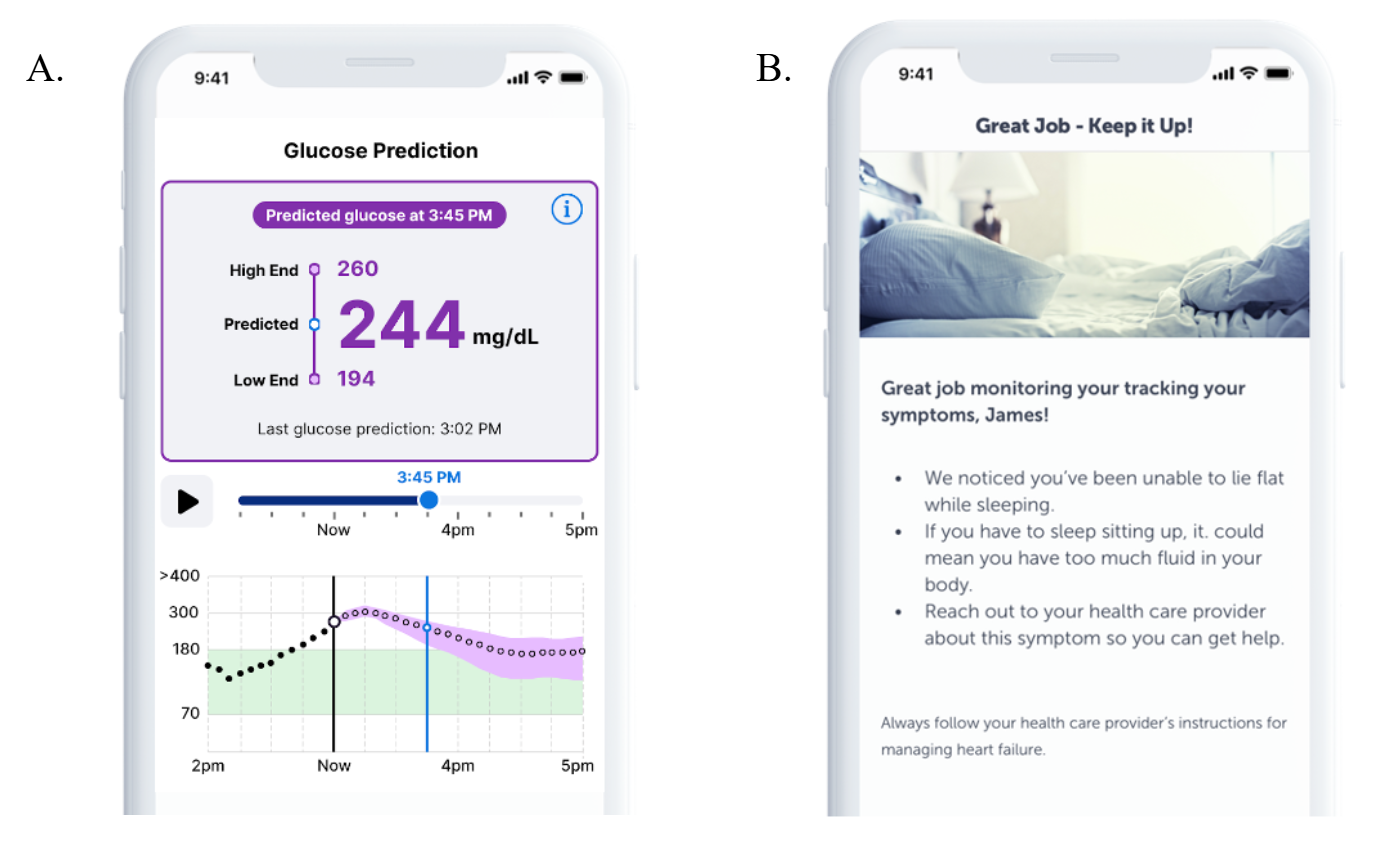

Supplement: Supplementary Figure 2 — User-generated health data and digital coaching. [file Image2.png]

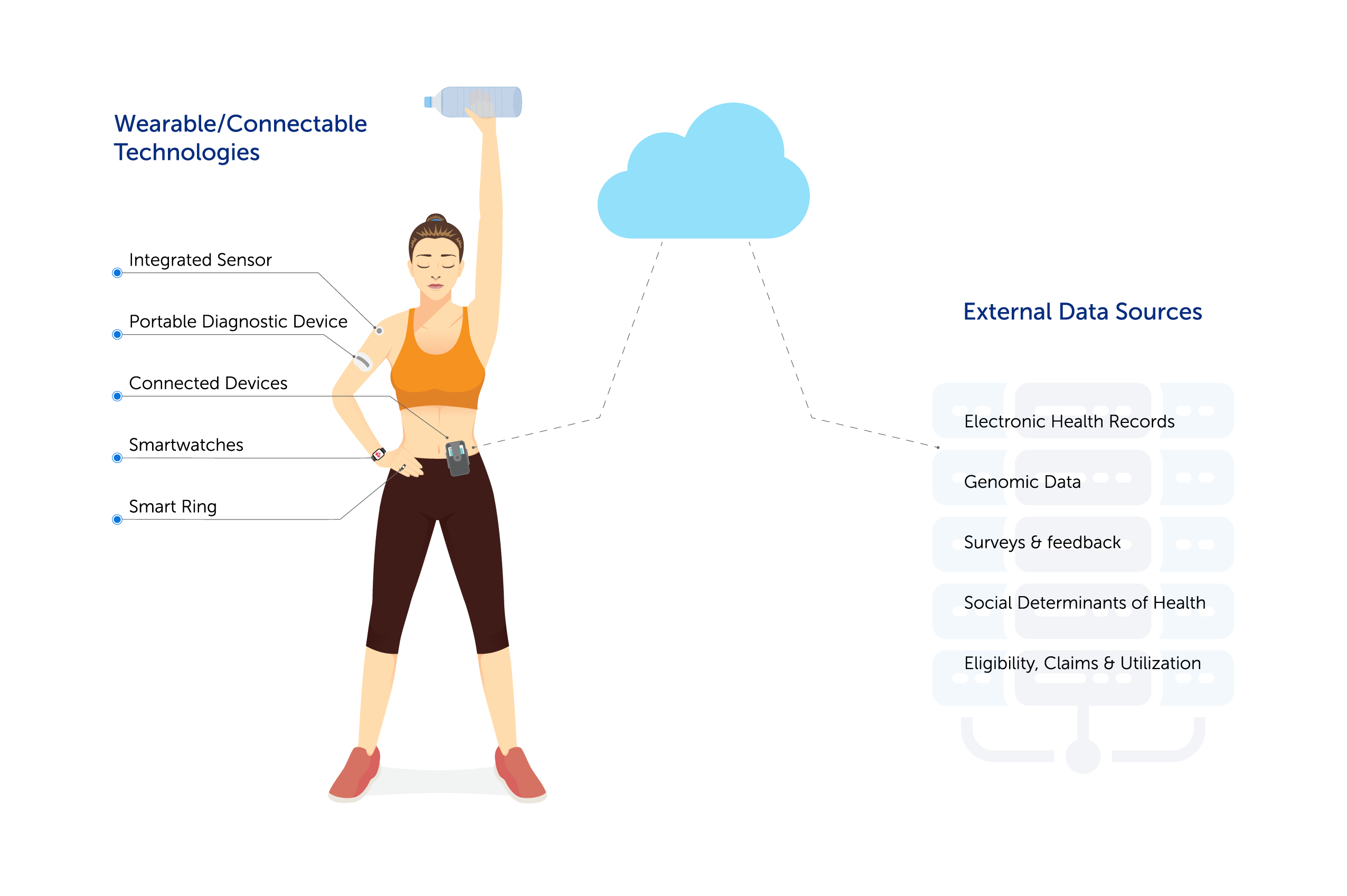

Supplement: Supplementary Figure 3 — Wearable/connectables technologies and external data sources. [file Image3.tif]

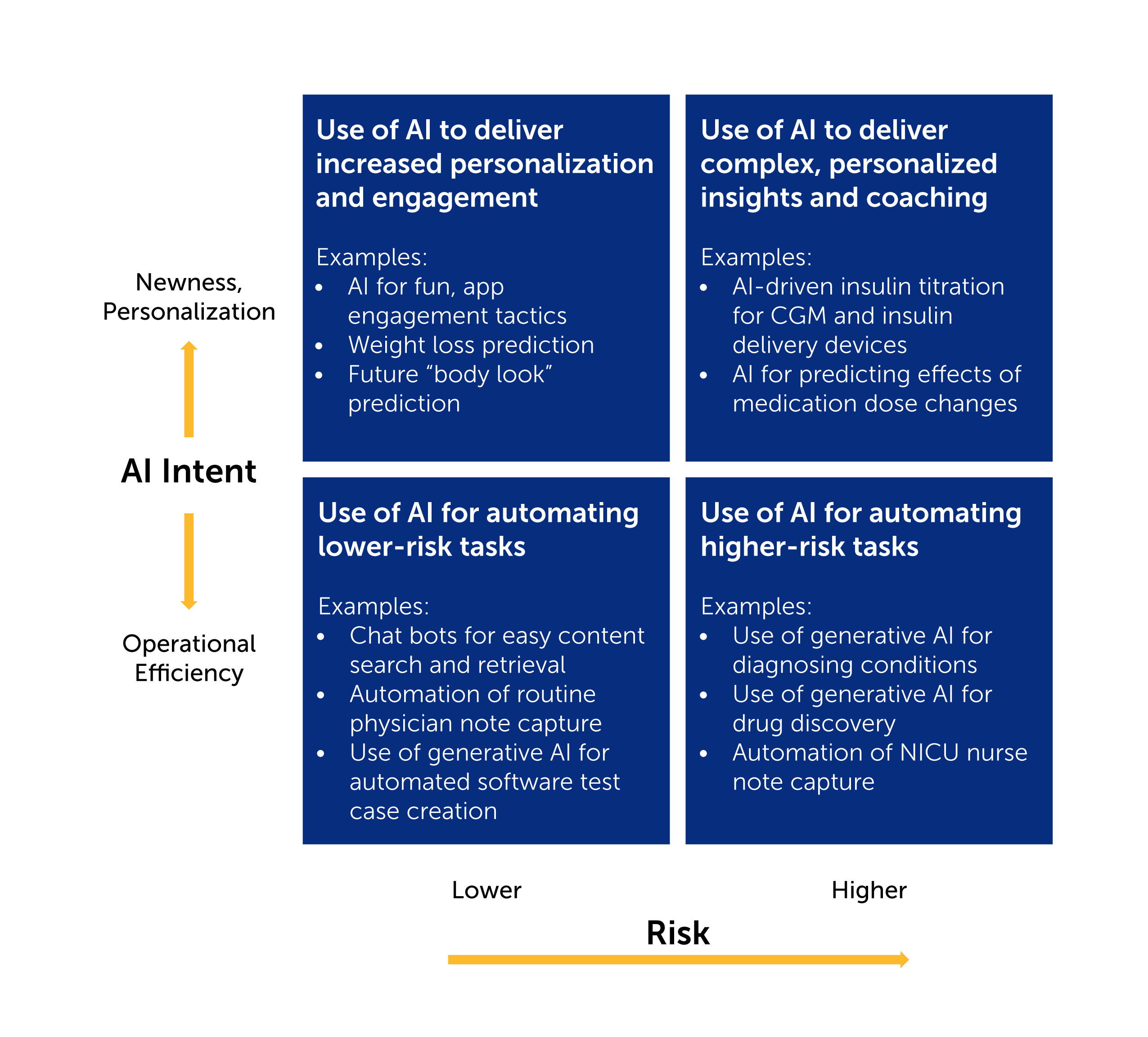

Supplement: Supplementary Figure 4 — Balancing AI intent with risk. [file Image4.tif]
